# Cellular distribution and function of ion channels involved in transport processes in rat tracheal epithelium

**DOI:** 10.14814/phy2.13290

**Published:** 2017-06-22

**Authors:** Anne Hahn, Johannes Faulhaber, Lalita Srisawang, Andreas Stortz, Johanna J Salomon, Marcus A Mall, Stephan Frings, Frank Möhrlen

**Affiliations:** ^1^ Department of Animal Molecular PhysiologyCentre of Organismal StudiesUniversity of HeidelbergHeidelbergGermany; ^2^ Department of Translational PulmonologyTranslational Lung Research Center Heidelberg (TLRC)German Center for Lung Research (DZL)University of HeidelbergHeidelbergGermany

**Keywords:** Anoctamin, chloride secretion, ion transport, tracheal epithelium

## Abstract

Transport of water and electrolytes in airway epithelia involves chloride‐selective ion channels, which are controlled either by cytosolic Ca^2+^ or by cAMP. The contributions of the two pathways to chloride transport differ among vertebrate species. Because rats are becoming more important as animal model for cystic fibrosis, we have examined how Ca^2+^‐ dependent and cAMP‐ dependent Cl^−^ secretion is organized in the rat tracheal epithelium. We examined the expression of the Ca^2+^‐gated Cl^−^ channel anoctamin 1 (ANO1), the cystic fibrosis transmembrane conductance regulator (CFTR) Cl^−^ channel, the epithelial Na^+^ channel ENaC, and the water channel aquaporin 5 (AQP5) in rat tracheal epithelium. The contribution of ANO1 channels to nucleotide‐stimulated Cl^−^ secretion was determined using the channel blocker *Ani9* in short‐circuit current recordings obtained from primary cultures of rat tracheal epithelial cells in Ussing chambers. We found that ANO1, CFTR and AQP5 proteins were expressed in nonciliated cells of the tracheal epithelium, whereas ENaC was expressed in ciliated cells. Among nonciliated cells, ANO1 occurred together with CFTR and Muc5b and, in addition, in a different cell type without CFTR and Muc5b. Bioelectrical studies with the ANO1‐blocker *Ani9* indicated that ANO1 mediated the secretory response to the nucleotide uridine‐5′‐triphosphate. Our data demonstrate that, in rat tracheal epithelium, Cl^−^ secretion and Na^+^ absorption are routed through different cell types, and that ANO1 channels form the molecular basis of Ca^2+^‐dependent Cl^−^ secretion in this tissue. These characteristic features of Cl^−^‐dependent secretion reveal similarities and distinct differences to secretory processes in human airways.

## Introduction

Secretion of electrolytes and water in airway epithelia is driven to a large extent by electrogenic Cl^−^ transport. The set of channels and transporters involved in this process has been studied extensively in human and murine epithelia of the nose, the trachea and the lung. Cl^−^ and fluid secretion is required to produce a thin layer of liquid covering the airway surfaces. Several types of Cl^−^ channels conduct anions across the apical membrane into the mucociliary layer that covers the airway surface. Cystic fibrosis transmembrane conductance regulator (CFTR) Cl^−^ channels are controlled by the cAMP‐signaling pathway (Pilewski and Frizzell 1999). Ca^2+^‐dependent Cl^−^ channels open when the cytosolic Ca^2+^ concentration increases and mediate secretory activity in response to purinergic stimulation. The Cl^−^ channel anoctamin 1 (ANO1) was proposed to fulfill this role in murine and in human airway epithelia (Ousingsawat et al. 2009; Rock et al. 2009; Jang and Oh 2014). Epithelial Na^+^ channels (ENaC) as the rate‐limiting pathway for ion/fluid absorption, aquaporin water channels and Cl^−^ channels together provide the persistent liquid cover of the airway surface. This air surface liquid (ASL) is required for normal mucociliary clearance, constituting an important innate defense mechanism in mammalian airways (Mall and Galietta 2015). Interestingly, experiments with heterologously expressed channel proteins suggested that the channels involved in secretion may interact and may regulate each other. There is, however, limited evidence for the relevance of such interactions in vivo for physiological and pathophysiological conditions (Mall et al. 1996; Wei et al. 1999; Kunzelmann et al. 2000; Billet and Hanrahan 2013; Kunzelmann and Mehta 2013). Direct interactions between channel proteins may occur inside airway epithelial cells that express more than one of the channel types. However, functional interactions may also result from the impact of Ca^2+^ on the cAMP signaling pathway or vice versa (Ahuja et al. 2014). In any case, it is important to know whether the ion channels involved in Cl^−^ secretion are indeed coexpressed in the same cells of the airway epithelia, or whether they reside in different cells types, such as ciliated cells and nonciliated cells. Electrophysiological studies on airway epithelia from humans, rodents, pigs, and dogs have brought out functional differences that can partly be interpreted in terms of different cellular expression patterns of ion channels (Hwang et al. 1996; Jiang and Engelhardt 1998; Kreda et al. 2005; Rogers et al. 2008; Enuka et al. 2012; Althaus 2013; Gianotti et al. 2016).

In the human lower airway epithelia, CFTR Cl^−^ channels are coexpressed with ENaC in ciliated cells (Kreda et al. 2005), while Ca^2+^‐gated Cl^−^ channels are expressed in nonciliated cells (Huang et al., 2012; Sondo et al. 2014; Caci et al. 2015). In rodents, the channels may show different expression patterns that could require modified concepts of epithelial secretion. Recently, a CFTR‐knockout rat has become available as a new animal model for cystic fibrosis (Tuggle et al. 2014). The respiratory system of rats is much larger than that of mice, has – like the human system – submucosal glands in the intrapulmonary airways, and presents various advantages for studies of physiology and pathophysiology. Thus, additional information on the molecular pathways of ion transport in rat airway epithelia is required for further studies. In this study, we examined the sites of expression of ENaC, CFTR, ANO1, and AQP5 in the rat tracheal epithelium to determine cell‐type distribution and coexpression. We asked, which of these proteins are coexpressed in the same cells, and may, therefore, contribute to transepithelial ion/fluid secretion and/or absorption in a coordinate manner. We specifically examined the function of ANO1 Cl^−^ channels in the tracheal epithelium, as Ca^2+^‐gated Cl^−^ channels are thought to play a dominant role in secretory processes in rodent airways (Hwang et al. 1996). We tested the contribution of ANO1 to Cl^−^ secretion in primary rat tracheal epithelial cell cultures using Ussing‐chamber recordings and the ANO1 inhibitor *Ani9* (Seo et al. 2016).

## Methods

### Animals

Wistar rats of both sexes (12–16 weeks) were obtained from Charles River Laboratories, Sulzfeld, Germany. The animals were housed in a pathogen‐free environment under standardized conditions. Food and water were provided ad libitum. Rats were killed either by increasing the concentration of CO_2_ or, for the isolation of primary tracheal epithelial cells, by intraperitoneal injection of an overdose of ketamine (300 mg/kg) and xylazine (15 mg/kg). All experiments conducted were approved by the Regierungspräsidium Karlsruhe and were conducted in agreement with national and international guidelines.

### Immunohistochemistry of airway epithelia

Tracheae were dissected from adult rats and fixed in paraformaldehyde (PFA, 4% w/v) in PBS (130 mmol/L NaCl, 8.1 mmol/L Na_2_HPO_4_, 1.9 mmol/L NaH_2_PO_4_, pH 7.4) for 2 h. The tissue was dehydrated in 10% sucrose for 2 h and cryoprotected in 30% sucrose overnight at 4°C. The specimens were embedded in Tissue Freezing Medium (Leica, Nussloch, Germany). Cryosections (20 *μ*m thick) were prepared on a cryostat at −22°C (CM3050 Leica Microsystems, Wetzlar, Germany). Sections were collected on gelatin‐covered glass slides (Superfrost, Carl Roth, Karlsruhe, Germany) and air‐dried. For immunostaining, the cryosections were first fixed for 15 min in 4% PFA and washed in PBST (0.5% v/v Tween 20 in PBS, pH 7.4). Sections were then incubated in SDS (1% w/v in PBS, pH 7.4) to unmask epitopes for antibody binding. This was followed by a 5‐min washing step in PBS and a 90‐min incubation in ChemiBLOCKER (Merck Millipore, Darmstadt, Germany) solution (CT solution: 20% v/v ChemiBLOCKER, 0.5% w/v Triton X‐100 in PBS, pH 7.4) to reduce background signals and facilitate antibody access. CT solution was then replaced with CTA solution (CT + 1% NaN_3_) containing the primary antibody. Overnight antibody incubation was followed by washing in PBST. Fluorescence‐tagged secondary antibodies were then incubated for 90 min in C solution (20% v/v ChemiBLOCKER in PBS, pH 7.4) and sections were again rinsed with PBST. For costainings, nuclei were stained with 0.3 *μ*mol/L 4,6‐diaminidino‐2‐phenylindole in PBS (DAPI; C‐7509; Life Technologies, Darmstadt, Germany). Sections were then embedded in nonfluorescent mounting medium (Aqua‐Poly/Mount, Polyscience, Eppelheim, Germany).

The following primary antibodies and dilutions were used: (1) polyclonal rabbit anti‐ANO1 (Abcam, ab53212, dilution 1:50) (2) polyclonal goat anti‐ANO1 (Santa Cruz, sc‐69343, dilution 1:50); (3) polyclonal rabbit anti‐CFTR (Alomone labs, ACL‐006; dilution 1:400); (4) monoclonal mouse anti‐CFTR (Cystic fibrosis foundation, no 570, dilution 1:80); (5) rabbit anti‐β‐ENaC was kindly provided by Dr. Alexei Diakov (Krueger et al. 2009) and diluted 1:200; (6) monoclonal mouse anti‐acetylated α‐tubulin (Sigma, T7451, dilution 1:100), (7) polyclonal rabbit anti‐Muc5b (Santa Cruz, sc‐20119, dilution 1:100); (8) polyclonal rabbit anti‐AQP5 (Santa Cruz, sc‐28628, dilution 1:100). The specificity of all antisera was demonstrated in previous publications, including (1) ANO1 (Dutta et al. 2011); (2) ANO1 (Dutta et al. 2011); (3) CFTR (Tabeling et al. 2015); (4) CFTR (Kreda et al. 2005); (5) β‐ENaC (Krueger et al. 2009). (6) Acetylated α‐tubulin was previously characterized as an immunohistochemical marker for ciliated airway epithelial cells (Scudieri et al. 2012); (7) Muc5b as a marker for a subpopulation of secretory goblet cells (Rousseau et al. 2003); (8) AQP5 (Dauner et al. 2012; Zhao et al. 2014). Sections were then incubated with a 1:1000 dilution of the respective AlexaFluor‐labeled F(ab)2 fragment (A‐111055, A‐21206, A‐11008, A‐11004, A‐11011, and A‐10042; Molecular Probes) in solution C. Control experiments without primary antibodies showed no signal (Fig. S1). All signals were analyzed using a Nikon C1 spectral imaging confocal laser scanning system.

### Cloning of rat ANO1

Rat nasal tissue was isolated using Dynabeads^®^ Magnetic Beads (Invitrogen; Life Technologies Darmstadt, Germany) by reverse transcription using random hexamer primers (Thermo Scientific, Pittsburgh) and SuperScript^®^ III Reverse Transcriptase (Invitrogen). Full length cloning of rat ANO1 (abc) in expression vector pEYFP‐N1 was performed as described in (Vocke et al. 2013).

### Heterologous expression and patch‐clamp analysis of ANO1‐blocker efficiency

Expression of ANO1‐pEYFP‐N1 in HEK293 cells was performed 48 h before the patch clamp experiments using the MATra‐A Reagent (PromoKine, Heidelberg, Germany) according to the manufacturer's instructions. As a reporter for transfection efficiency, we used a previously described recombinant fusion construct carrying a yellow fluorescent protein tag (Vocke et al. 2013). Plasma‐membrane localization was checked using tetramethylrhodamine‐conjugated wheat germ agglutinin (Molecular Probes, W849). For patch clamp recordings, HEK293 cells were grown on poly‐L‐lysine‐coated glass coverslips, which were transferred to the recording chamber on the stage of an upright Nikon Eclipse microscope. All experiments were performed in CsCl solution to inhibit Ca^2+^‐dependent cation currents. The bath solution contained 150 mmol/L CsCl, 10 mmol/L HEPES and 10 mmol/L EGTA (pH 7.4 with CsOH). Pipette solutions containing defined concentrations of free Ca^2+^ were prepared according to (Reisert et al. 2003). All pipette solutions contained 140 mmol/L CsCl, 10 mmol/L HEDTA and 10 mmol/L HEPES. Depending on the desired concentration of free Ca^2+^ ions, CaCl_2_ was added (in mmol/L: 1.1232 mmol/L for 0.25 *μ*mol/L free Ca^2+^, 3.209 mmol/L for 0.75 *μ*mol/L‐free Ca^2+^ and 5.866 for 2.4 *μ*mol/L‐free Ca^2+^, pH 7.2). Cells were selected for similar size, general healthy appearance and similar intensity of YFP fluorescence to provide similar levels of protein expression. On‐cell gigaseals were established using borosilicate capillaries (outer diameter 1.5 mm, inner diameter 0.86 mm) with a resistance of 2.5–3.5 MΩ connected to a patch clamp amplifier (EPC‐8, HEKA Elektronik, Lambrecht/Pfalz, Germany). For continuous current recordings, the holding potential was set to −70 mV. After reaching a pipette gigaseal of at least 1.5 GΩ, whole‐cell conformation was established and the pipette current was monitored for 60 sec. In addition, whole‐cell current‐to‐voltage relations were constructed from 1‐s voltage ramps with 0.75 *μ*mol/L Ca^2+^ in the pipette solution. Data were low‐pass filtered at 3 kHz at 10 kHz sampling speed (BNC 2120; National Instruments, Austin TX) using the electrophysiology software WinWCP provided by the University of Strathclyde (Glasgow, Scotland, UK).

### Primary culture of RTEC

Dissociation and culture of rat tracheal epithelial cells (RTEC) was performed according to published protocols (Kaartinen et al. 1993; Davidson et al. 2000). Rats were killed by intraperitoneal injection of ketamine and xylazine. After opening the abdominal cavity, animals were exsanguinated by cutting the *Vena cava*. The trachea was freed from the esophagus and surrounding muscle, vessels and connective tissue. It was cut open longitudinally and, after removal from the body, shortly washed in collection medium (500 mL DMEM/F12; Thermo Fisher/Gibco 21331‐020; with 5 mL Pen/Strep 100x; GIBCO #15140‐122). Afterwards, the tissue of each animal was incubated overnight at 4°C in 20 mL dissociation medium containing Protease E (20 mg/mL, Sigma, P8811‐1G) and DNaseI (10 mg/mL SERVA, 18535). The dissociation medium was 500 mL PBS containing 1.8 g NaHCO_3_ (Sigma, 31437), 2.5 *μ*L FeN_3_O_9_ (0.25 *μ*mol/L, Sigma, F8508), 5 *μ*L Na‐pyruvate (100 mmol/L, Sigma, S8636), 3 mL Pen/Strep. (100x). To 20 mL of this medium, 200 *μ*L DNaseI Stock solution (10 mg/mL) and 1.4 mL protease E stock solution were freshly added for each cell isolation.

On the following day, the trachea was incubated for 1 h at 37°C in the dissociation medium. Afterwards the dissociation reaction was stopped by adding 5 mL heat inactivated fetal bovine serum (HI‐FBS, Sigma, F9665) and the cell solution was filtered through a 100 *μ*m gauge cell strainer. To isolate fibroblasts from the preparation, the cells were incubated for 2.5 h at 37°C on plastic dishes. Epithelial cells did not attach to the dish during this incubation time, were removed, counted and seeded at a density of 600.000 cells per filter on Transwell (3460) or Snapwell (3407) (Corning, Tewksbury MA) permeable filter inserts. Cells were cultured, using DMEM/F12 (1:1) medium containing the following components of the Clonetics SAGM Single Quot kit (Lonza, Basel, Switzerland; CC‐4124): bovine serum albumin, bovine pituitary extract, insulin, transferrin, hydrocortisone, human recombinant epidermal growth factor and retinoic acid. Additionally, the culture medium was supplemented with 2 mmol/L l‐glutamine (Sigma, G7513), 0.5 mg/mL Primocin (Invitrogen; ANTPM1) as well as 0.1 *μ*g/mL cholera toxin (Sigma, C8052). Ten percent HI‐FBS was added to the medium in the basolateral compartment for the first 24 h after seeding. After 24 h, the basolateral medium was replaced by culture medium containing 1% BSA. From day 3 on, the cells were cultured under air–liquid culture conditions. The basolateral medium was changed every second day. Each experiment with RTEC cultures was performed with cells from at least 4 different cell isolations.

### Fura‐2 Ca^2+^ Imaging of RTEC cultures

Primary rat tracheal epithelial cells grown on Transwell permeable filter inserts for at least 14 days were used for Ca^2+^‐imaging analyses. 50 *μ*g of fura‐2‐AM (Invitrogen, F1221) were solved in 10 *μ*L Pluronic F‐127 (Thermo Fisher Scientific, P3000MP). The cells were incubated with 4 *μ*mol/L fura‐2 in 500 *μ*L isotonic Ringer buffer (in mmol/L: 145 NaCl, 0.4 KH_2_PO_4_, 1.6 K_2_HPO_4_, 5 Glucose, 1 MgCl_2_, 1.3 Ca‐Gluconate; pH 7.4) for 45 min at 37°C. Afterwards they were washed with Ringer buffer for another 20 min at 37°C. The filter inserts were placed on glass bottom dishes, and fluorescence images were taken using an inverted microscope (Nikon ECLIPSE Ti fluorescence microscope) with a Polychrome V monochromator (FEI Munich, Germany) and a CoolSnap CCD camera (Photometrics, Tuscon AZ). Each Ca^2+^ imaging experiment was monitored for 5 min (1 frame/sec with an exposure time of 50 msec and 2 × 2 binning). After 1 min, 100 *μ*mol/L UTP was applied to trigger intracellular Ca^2+^ release. To determine the absolute intracellular Ca^2+^ concentration after UTP stimulation, calibration experiments for fluorescence ratios R = F_340_/F_380_ were performed according to (Grynkiewicz et al. 1985). Cells were either permeabilized with 10 *μ*mol/L ionomycin (Sigma, I3909) to achieve a complete saturation of intracellular fura‐2 with Ca^2+^ (*R*_max_), or they were incubated with Ca^2+^‐free buffer for 20 min before the measurement to determine the fura‐2 fluorescence level under Ca^2+^‐free conditions (*R*_min_). Calibrated Ca^2+^ concentrations were calculated according to [Ca^2+^] = *K*_D_ (*R*‐*R*_min_)/(*R*_max_‐*R*)*(F_380_free/F_380_bound) with *K*_D_ = 145 nmol/L. To test whether the ANO1 inhibitor *Ani9* affected UTP‐induced Ca^2+^ signals, cells were preincubated with 10 *μ*mol/L *Ani9* for 5 min before application of UTP.

### Short‐circuit current recording from RTEC cultures

Bioelectrical short‐circuit current measurements were performed in EasyMount Ussing chambers (Physiologic Instruments, San Diego CA) as previously described (Salomon et al. 2016). Rat primary tracheal epithelial cells grown on Snapwell permeable filter inserts for at least 14 days were mounted into Ussing chambers. Both sides were filled with Ringer buffer solution (described above). Amiloride (100 *μ*mol/L) was added to the apical side to inhibit the Na^+^ conductance. IBMX (3‐isobutyl‐1‐methylxanthine, Sigma, I7018, 100 *μ*mol/L) and FSK (forskolin, Sigma, F3917, 1 *μ*mol/L,) were added to both filter sides to increase intracellular cAMP levels. CFTR_inh_172 (Sigma, C2992, 20 *μ*mol/L) was added to the apical side to inhibit CFTR‐conductance. To increase intracellular Ca^2+^ levels, UTP (uridine‐5′‐triphosphate, Sigma, U1006, 100 *μ*mol/L) was added to the apical side. In a subset of experiments, Ringer buffer was replaced by 5 mmol/L Cl^−^ gluconate buffer (in mmol/L: 5 NaCl, 0.4 KH_2_PO_4_, 1.6 K_2_HPO_4_, 5 Glucose, 1 MgCl_2_, 140 Na‐gluconate, 8 Ca‐gluconate; pH 7.4) on both sides. UTP (100 *μ*mol/L) was applied to the apical side to analyze the UTP‐induced currents in a low Cl^−^ environment. Subsequently, both sides of the chambers were washed once with Ringer buffer and then filled with Ringer buffer again. UTP (100 *μ*mol/L) was applied again to determine whether UTP‐induced currents are dependent on the Cl^−^ concentration in the buffer. To further characterize UTP‐induced currents, the general Cl^−^ channel blocker niflumic acid (Sigma, N0630, 500 *μ*mol/L) was added to the apical side before IBMX/FSK and UTP application. In another set of experiments, the ANO1‐inhibitor *Ani9* (10 *μ*mol/L; Innovapharm, Kiev, Ukraine) was added to the apical side either before IBMX/FSK or before UTP application.

### Statistical analysis

Statistics were calculated and the graphs were prepared using OriginLab 9.0 (OriginLab Corporation, Northampton). Data were first assessed for normal distribution using the Shapiro–Wilk test. All data were normally distributed, and a standard two‐tailed unpaired student's *t*‐test was calculated. As a predetermined significance threshold, a *P* < 0.05 was determined (*P* < 0.05*; *P* < 0.01**; *P* < 0.001***). All data, if not indicated otherwise, are displayed as means ± SEM.

## Results

### Cellular distribution of epithelial ion channels in rat tracheal epithelium

**Figure 1:**
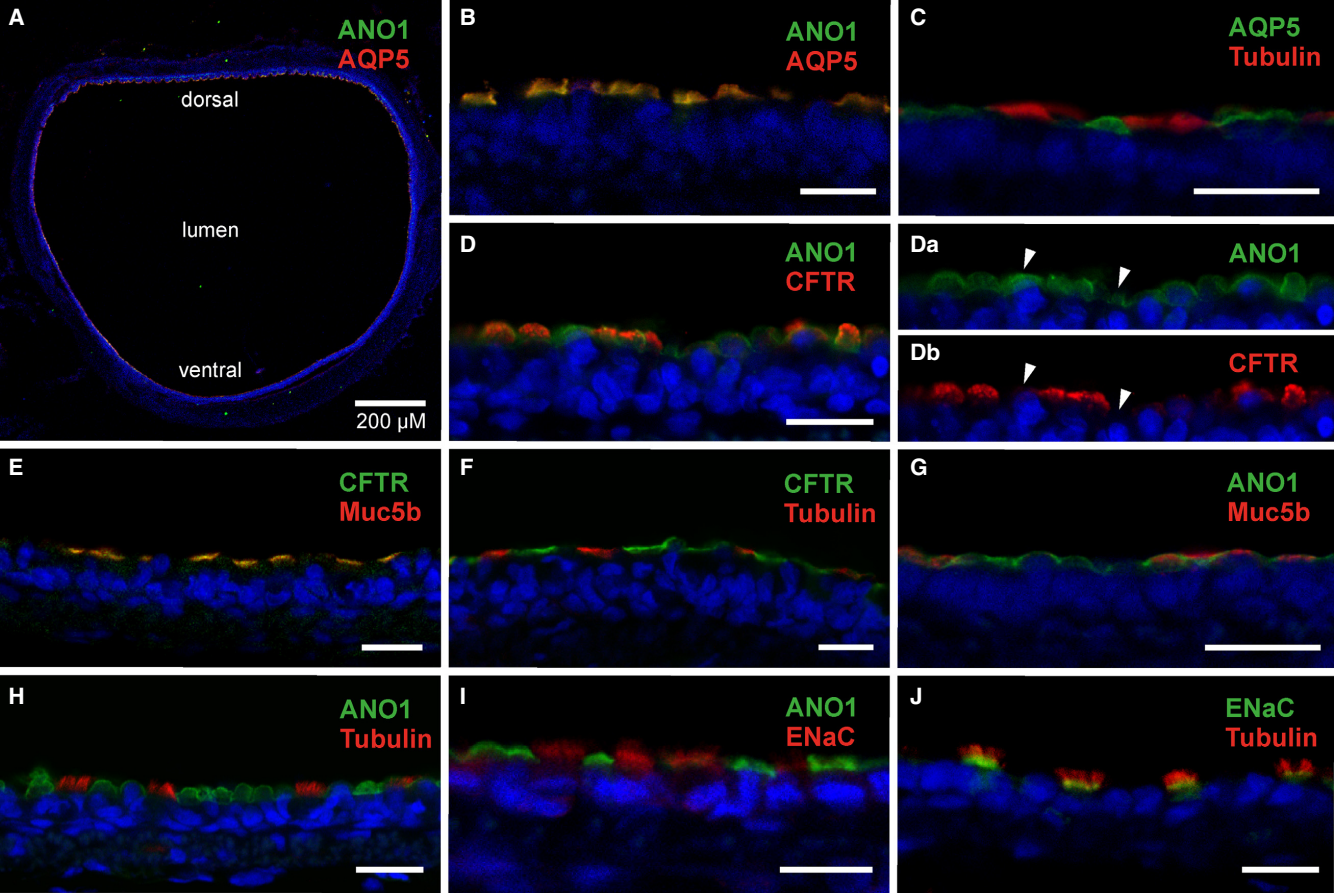
Cellular localization of secretory proteins in rat tracheal epithelium. (A) A cross‐section of the medial part of the rat trachea with immunosignals for ANO1 and AQP5 at the apical surface of the tracheal epithelium. (B) Immunosignals of ANO1 and AQP5 merge in cells stained in the surface epithelium. (C) AQP5 is not expressed in *α*‐tubulin‐positive ciliated cells. (D) ANO1‐ and CFTR‐immunosignals merge in most cells, but some show only ANO1 signals. (Da)*,* (Db) Separate display of ANO1‐ and CFTR‐ immunofluorescence channels illustrates that some ANO1‐positive cells are CFTR‐negative *(arrowheads)*. (E) CFTR‐ and Muc5b‐ immunosignals merge to produce a yellow signal in all stained cells. (F) CFTR‐ and α‐tubulin‐immunostainings label different cells, no merged signals are discernible. (G) ANO1 and Muc5b immunosignals appear colocalized in some cells, but other ANO1‐positive cells are Muc5b‐negative. (H) ANO1 is absent from *α*‐tubulin‐positive ciliated cells. (I) ANO1‐ and ENaC‐ immunosignals are localized in different cell types. No merged signal is discernible. (J) The ENaC‐specific immunosignal emanates from a distinct layer at the basal region of α‐tubulin‐expressing cilia. Blue signals show DAPI nuclear stain. Scale bars for B–J indicate 10 *μ*m.

To assess the expression sites of proteins involved in epithelial transport (ANO1, AQP5, CFTR, ENaC), we used marker proteins to identify individual cell types in the tracheal epithelium (acetylated α‐tubulin for ciliated cells and Muc5b for nonciliated cells). Cryosections were obtained from the lateral or ventral regions of the rat trachea, to examine expression patterns in the surface epithelium (Fig. 1A). ANO1 channel protein was colocalized with AQP5 in apical surface structures of tracheal epithelial cells (Fig. 1B). These cells did not express α‐tubulin, a marker for ciliated cells (Fig. 1C), indicating that only nonciliated cells express ANO1 and AQP5. In costainings of the two Cl^−^ channels ANO1 and CFTR, many cells were found to express both proteins (Fig. 1D), but a subpopulation of cells clearly expressed only ANO1, and not CFTR (Fig. 1Da, Db). CFTR‐positive cells, in turn, always expressed ANO1. Thus, in the rat trachea, both ANO1 and CFTR appear to be expressed exclusively in nonciliated cells, but not always together. To characterize the nonciliated cells that express ANO1 and CFTR Cl^−^ channels, we costained against Muc5b, a marker for secretory goblet cells. The CFTR immunosignal was always colocalized with Muc5b (Fig. 1E). This result suggests a specific expression of CFTR in nonciliated, secretory goblet cells of the rat tracheal epithelium, a notion corroborated by the mutually exclusive expression patterns of CFTR and *α*‐tubulin (Fig. 1F). In contrast to this strict colocalization, ANO1 and Muc5b were coexpressed by some, but not by all, epithelial cells (Fig. 1G). As Muc5b‐negative cells are also CFTR‐negative, there must be a population of nonciliated tracheal cells that expresses ANO1 and AQP5, but neither CFTR nor Muc5b. This cell type may represent club cells, a distinct population of nonciliated cells residing in the lower airways (Tokita et al. 2014). ANO1 was absent from *α*‐tubulin‐positive ciliated cells (Fig. 1H) and also from ENaC‐expressing cells (Fig. 1I). This finding demonstrated a restricted expression of ENaC proteins in ciliated cells, demonstrated also by costaining with α‐tubulin (Fig. 1J). ENaC can be seen in an apical layer just underneath the *α*‐tubulin‐stained cilia, which probably represents the microvillar layer of the ciliated cells. Taken together, these data demonstrate a distinct expression pattern of the investigated proteins in three epithelial cell types of the rat trachea. (1) ciliated cells express ENaC, but neither ANO1 nor CFTR or AQP5; (2) nonciliated, Muc5b‐positive cells coexpress ANO1, CFTR and AQP5, but no ENaC; and (3) nonciliated, Muc5b‐negative cells express ANO1 and AQP5, but neither CFTR nor ENaC.

### Ion transport properties of primary tracheal epithelial cell cultures from rats

**Figure 2:**
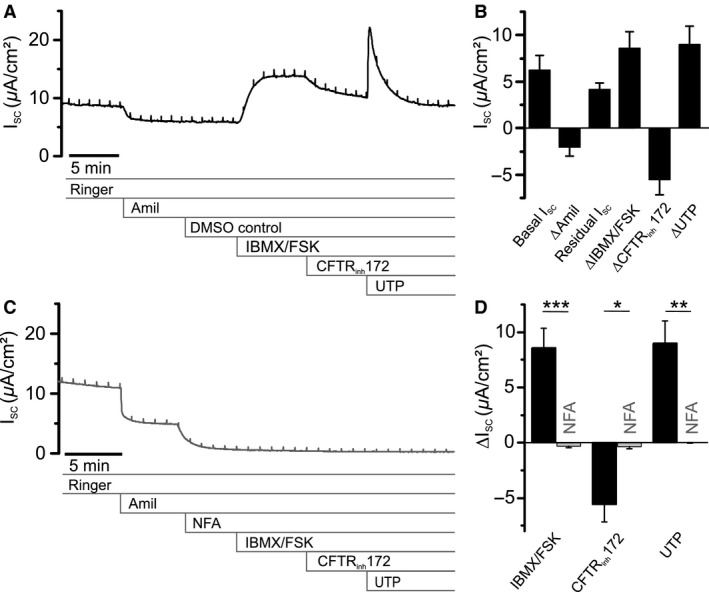
Short‐circuit currents associated with secretory activity in primary cultures of rat tracheal epithelia cells (RTEC). (A) Representative short‐circuit current (*I*_SC_) trace from RTEC monolayers grown on permeable filter supports recorded in an Ussing chamber. Shown are the inhibitory effects of amiloride (Amil, 100 *μ*mol/L, apical) and CFTR_inh_172 (20 *μ*mol/L, apical), as well as the activating compounds 3‐isobutyl‐1‐methylxanthine (IBMX, 100 *μ*mol/L, basolateral and apical), forskolin (FSK, 1 *μ*mol/L, basolateral and apical) and uridine‐5′‐triphosphate (UTP, 100 *μ*mol/L, apical). (B) Statistical summary of the basal *I*_SC_ and of the effects of the test compounds. The residual, amiloride‐insensitive current accounts for most of the basal *I*_SC_. Application of IBMX/FSK and UTP causes substantial increase in *I*_SC_, while CFTR_inh_172 reduces the signal. (C) Representative trace displaying the effects of amiloride (Amil, 100 *μ*mol/L, apical) and niflumic acid (NFA, 500 *μ*mol/L, apical) on *I*_SC_ in RTEC monolayers. The residual *I*_SC_ in the presence of amiloride was significantly reduced by NFA (*P *<* *0.05). (D) Statistical summary of *C* with degree of inhibition by NFA of Δ*I*_SC_ induced by IBMX/FSK, CFTR_inh_172 and UTP. All results are means ± SEM (*n* = 5 primary cultures). *P <* *0.05, **P <* *0.01, ***P <* *0.001 (two tailed) between cultures exposed to test compounds and controls.

**Figure 3:**
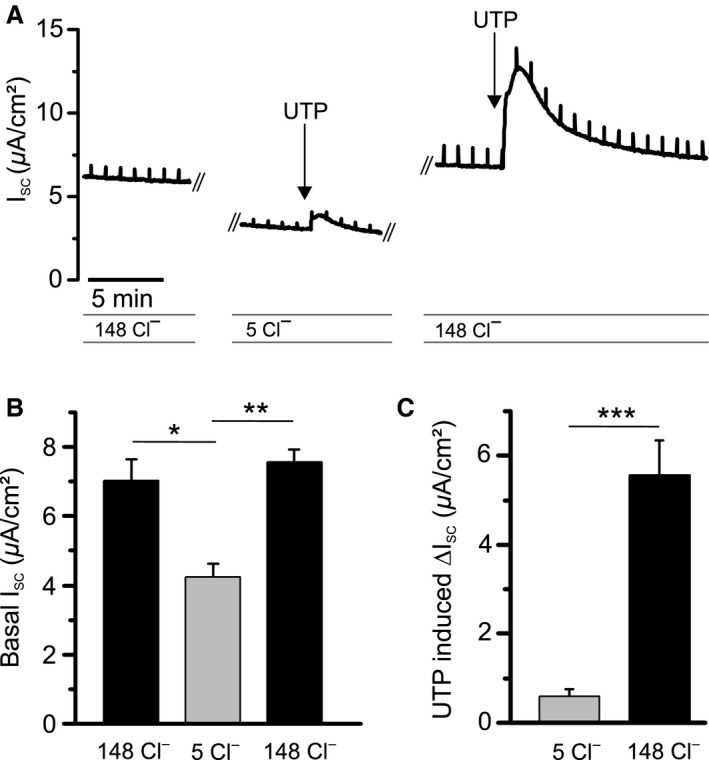
Cl^−^‐dependence of UTP‐induced Δ*I*_SC_ responses in RTEC monolayers. (A) Representative *I*_SC_ trace demonstrating the effect of Cl^−^ substitution by gluconate on basal *I*_SC_ as well as on the UTP‐induced Δ*I*_SC_ response. Cl^−^ concentrations are indicated in mmol/L. (B) Statistical summary of basal *I*_SC_ upon reducing Cl^−^ concentrations on both sides of the monolayer, and recovery upon return to high Cl^−^ solution. (C) Statistical summary of the effect of Cl^−^ substitution on the UTP‐response. All results are means ± SEM (*n* = 4 primary cultures). *P <* *0.05, **P <* *0.01, ***P <* *0.001 (two tailed) between cultures exposed to test compounds and controls.

Since Ca^2+^‐dependent Cl^−^ secretion was suggested to play a more prevalent role in rodent airways compared to humans, we examined this pathway functionally. To quantify the contribution of ANO1 channels to apical Cl^−^ secretion by rat airway epithelial cells, a primary tracheal epithelial cell (RTEC) culture was used (Kaartinen et al. 1993; Hwang et al. 1996). Epithelial cells obtained from a rat trachea formed a monolayer on permeable filter supports, with tight junctions connecting individual cells. Cultures were used for bioelectrical experiments in Ussing chambers (Fig. 2A) after being grown on Snapwell^TM^ permeable filter inserts for at least 14 days. These cultures showed an average transepithelial resistance of 1640 Ωcm^2^ and a basal short circuit current *I*_SC_ of 6.22 ± 1.55 *μ*A/cm^2^ (*n* = 5). Application of the ENaC blocker amiloride (100 *μ*mol/L, Fig. 2B) revealed ENaC‐mediated Na^+^ absorption (Δ*I*_SC_ = −2.07 ± 0.95 *μ*A/cm^2^; *n* = 5). cAMP‐dependent transport pathways were activated by application of 1 *μ*mol/L forskolin in the presence of 100 *μ*mol/L IBMX to both sides of the epithelial monolayer. This protocol is designed to raise the cytosolic cAMP concentration and caused an increase Δ*I*_SC_ of 8.59 ± 1.75 *μ*A/cm^2^ (*n* = 5, Fig. 2B). The subsequent addition of 20 *μ*mol/L CFTR inhibitor CFTR_inh_172 (Ma et al. 2002; Gianotti et al. 2016) to the apical solution reduced *I*_SC_ by −5.58 ± 1.59 *μ*A/cm^2^ (*n* = 5, Fig. 2B), demonstrating that this current was mediated by CFTR. Finally, Ca^2+^‐dependent Cl^−^ secretion was triggered by 100 *μ*mol/L UTP that was added to the apical solution and produced a transient increase in short‐circuit current with a peak Δ*I*_SC_ of 9.0 ± 1.95 *μ*A/cm^2^ (*n* = 5, Fig. 2B). To verify that cAMP‐dependent and Ca^2+^‐dependent responses were caused by Cl^−^ channels, we repeated the experiments in the presence of the broad‐spectrum Cl^−^‐channel blocker niflumic acid (NFA, Fig. 2C). Application of 500 *μ*mol/L NFA to the apical solution in the presence of amiloride further decreased *I*_SC_ from 3.63 ± 0.84 *μ*A/cm^2^ to 1.26 ± 0.5 *μ*A/cm^2^ (*n* = 5, Fig. 2C), apparently by inhibiting a basal Cl^−^ secretion pathway. Moreover, NFA completely blocked the responses to IBMX/FSK (Δ*I*_SC_ = −0.33 ± 0.11 *μ*A/cm^2^) and to UTP (Δ*I*_SC_ = −0.00 ± 0.02 *μ*A/cm^2^) (*n* = 5; Fig. 2C, D). The identification of Cl^−^ as the charge carrier of the UTP‐induced *I*_SC_ transient was corroborated by an ion exchange experiment, where Cl^−^ was replaced by the impermeable anion gluconate in both compartments of the Ussing chamber (Fig. 3A). Basal *I*_SC_ decreased from 7.56 ± 0.37 *μ*A/cm^2^ at high Cl^−^ to 4.26 ± 0.34 *μ*A/cm^2^ at low Cl^−^ (*P *<* *0.01; *n* = 4) (Fig. 3B). The peak increase in *I*_SC_ triggered by UTP was reduced by 86% upon reducing Cl^−^ from 148 mmol/L to 5 mmol/L. Mean Δ*I*_SC_ was 5.57 ± 0.3 *μ*A/cm^2^ at high Cl^−^ and 0.76 ± 0.07 *μ*A/cm^2^ at low Cl^−^; *P *<* *0.001; *n* = 4) (Fig. 3C) suggesting that UTP‐mediated currents were carried by Cl^−^ secretion, as expected from previous studies (Hwang et al. 1996).

### ANO1‐mediated currents are blocked by *Ani9*

**Figure 4:**
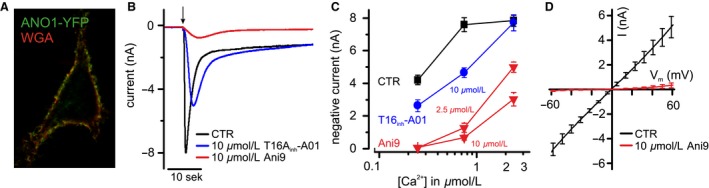
Inhibition of ANO1 Cl^−^ channels by *Ani9*. (A) ANO1‐YFP fusion protein is targeted to the plasma membrane of HEK293 cells. The cell surface is labeled with the lectin WGA, conjugated to a fluorescent dye. (B) Representative traces of Ca^2+^‐induced Cl^−^ currents in *Ano1*‐*YFP*‐transfected HEK293 cells with and without the Cl^−^ ‐channel blockers *T16A*_*inh*_ *‐A01* and *Ani9*. Whole‐cell currents were recorded at ‐70 mV with 0.75 *μ*mol/L Ca^2+^ in the pipette solution following formation of the whole‐cell configuration *(arrow)*. (C) Statistical summary of experiments in HEK293 cells, illustrating that the blocking efficiency depended on the intracellular Ca^2+^ concentration. Data are means ± SEM (*n* = 9–17 HEK293 cells per value). (D) Whole‐cell current‐to‐voltage relation of ANO1‐mediated Cl^−^ currents at 0.75 *μ*mol/L intracellular Ca^2+^ and block by 10 *μ*mol/L extracellular *Ani9*. Currents from 10 to 12 HEK293 cells were averaged, and background currents at 0 Ca^2+^ were subtracted.

To examine the question whether ANO1 underlies the UTP‐induced response in RTEC cultures, an efficient ANO1 inhibitor had to be identified. Recently, the compound *Ani9* emerged from a small‐molecule screen of Cl^−^ channel inhibitors as the first blocker for ANO1 channels that discriminates between ANO1 and the closely related ANO2 channel. *Ani9* was also reported to not inhibit CFTR channels (Seo et al. 2016). Before applying *Ani9* to RTECs in Ussing chambers, we assessed its blocking efficiency in ANO1‐transfected HEK293 cells. We first compared the effect of this compound with the ANO1 blocker *T16A*_*inh*_ *‐A01* (Namkung et al. 2011) using the ANO1‐channel splice variant ANO1abc that is expressed in airway epithelia (Caputo et al. 2008). ANO1abc was heterologously expressed in HEK293 cells for characterization and was found to be targeted to the plasma membrane (Fig. 4A). Whole‐cell currents were recorded from transfected cells with 0.25, 0.75, or 2.4 *μ*mol/L Ca^2+^ in the pipette solution. *Ani9* (10 *μ*mol/L) effectively suppressed ANO1‐mediated currents, whereas 10 *μ*mol/L *T16A*_*inh*_ *‐A01* had a much weaker effect (Fig. 4B). According to a recent report (Sung et al. 2016), the blocking efficiency of *T16A*_*inh*_ *‐A01* on ANO1 channels diminishes at increased cytosolic Ca^2+^ levels. To find out whether this Ca^2+^ interaction also applied to *Ani9*, we measured the blocking effects of 2.5 *μ*mol/L and 10 *μ*mol/L *Ani9* and 10 *μ*mol/L *T16A*_*inh*_ *‐A01* at different intracellular Ca^2+^ concentrations. It turned out that the blocking efficiency of both compounds was reduced when Ca^2+^ was raised over 1 *μ*mol/L (Fig. 4C). *Ani9* blocked with higher efficiency than *T16A*_*inh*_ *‐A01* at all Ca^2+^ concentrations, and *Ani9* block showed little voltage‐dependence (Fig. 4D). The Ca^2+^‐dependence of *Ani9* block indicates that the peak intracellular Ca^2+^ concentration during the UTP‐induced signal in RTEC cultures should be taken into account when selecting an effective *Ani9*‐concentration for ANO1‐blockage.

**Figure 5:**
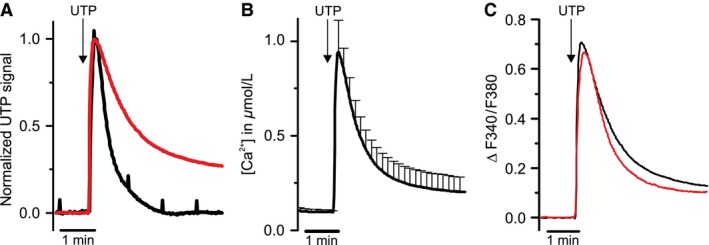
Intracellular Ca^2+^‐release during UTP stimulation in RTEC cultures. (A) Comparison of the time course of UTP‐induced Ca^2+^ signals recorded from RTEC cultures *(red trace*, normalized averaged traces from 4 primary cultures) with a representative UTP‐induced Δ*I*_SC_ signal recorded from RTEC cultures in the Ussing chamber *(black trace*). (B) Calibrated Ca^2+^ imaging traces from RTEC cultures visualize the intracellular Ca^2+^ concentration during stimulation with UTP (100 *μ*mol/L) (mean ± SEM, averaged traces from 4 primary cultures). (C) Comparison of the intracellular Ca^2+^‐response of RTEC cultures during UTP‐stimulation with *(red trace)* and without *(black trace) Ani9* (10 *μ*mol/L) (averaged traces from 4 primary cultures).

To determine the peak Ca^2+^ signal produced by RTEC cultures during UTP‐stimulation, we used *fura‐2* Ca^2+^ imaging on RTECs grown on Transwell^®^ permeable filter inserts for at least 14 days. UTP‐induced Ca^2+^ signals in RTEC cultures showed a similar onset speed as *I*_SC_ recordings from the Ussing chamber (Fig. 5A). Using calibrated *fura‐2* signals, we obtained estimates for the absolute values of intracellular Ca^2+^ concentrations, indicating a rise from below 0.1 *μ*mol/L to 0.95 ± 0.17 *μ*mol/L Ca^2+^ upon application of 100 *μ*mol/L UTP (Fig. 5B). At that Ca^2+^ concentration, an *Ani9* concentration of 10 *μ*mol/L is sufficient to block ANO1abc channels by over 90% (*cf*. Fig. 4C, D). Importantly, 10 *μ*mol/L *Ani9* did not significantly change amplitude or time course of the UTP‐induced Ca^2+^ signal in RTEC cultures (Fig. 5C), demonstrating its suitability as a specific blocker of ANO1 channels in Ussing‐chamber experiments.

**Figure 6:**
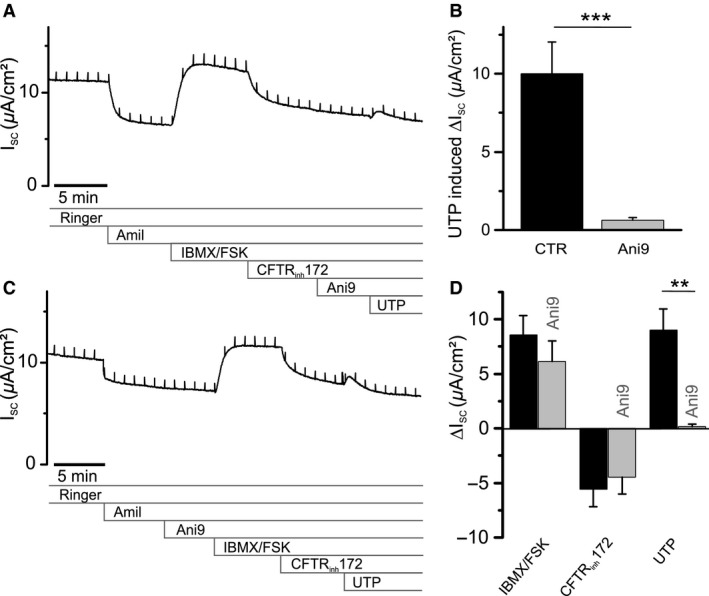
Pharmacological identification of Ca^2+^‐dependent Cl^−^ channels mediating UTP response in RTEC cultures. (A) Representative *I*_SC_ recording from an RTEC monolayer, with *Ani9* (10 *μ*mol/L) applied to the apical solution 5 min before stimulation with UTP (100 *μ*mol/L). (B) Statistical summary of (A) and control experiments without *Ani9,* illustrating blocking efficiency of 10 *μ*mol/L *Ani9* on the UTP response (*n* = 7 primary cultures). (C) Representative *I*_SC_ recording with 10 *μ*mol/L *Ani9* applied to the apical solution 5 min before stimulating the RTEC monolayer with IBMX/FSK, applying the protocol depicted in Fig. 2A. (D) Statistical summary of (C) and control experiments without *Ani9,* to show Δ*I*_SC_ responses of RTEC monolayers to the application of IBMX/FSK, CFTR_inh_172 and UTP in the presence of 10 *μ*mol/L *Ani9* (*n* = 5 primary cultures). All results are means ± SEM. *P <* *0.05, **P <* *0.01, ***P <* *0.001 (two tailed) between cultures exposed to test compounds and controls.

To test whether 10 *μ*mol/L *Ani9* would block UTP‐induced signals in RTEC cultures during *I*_SC_ recordings in the Ussing chamber, we added the compound to the apical solution 5 min before UTP application (Fig. 6A). *Ani9* almost completely blocked the subsequent response to UTP (Δ*I*_SC_ without *Ani9*: 10.02 ± 2.03 *μ*A/cm^2^, with *Ani9*: 0.63 ± 0.16 *μ*A/cm^2^; *P *<* *0.001; *n* = 7) (Fig. 6B). Since *Ani9* was reported to be ineffective on CFTR channels (Seo et al. 2016), we tested this on the RTEC cultures. In a new set of experiments, we applied *Ani9* to the apical solution before IBMX/FSK (Fig. 6C). We found that the compound did not significantly change the cAMP‐dependent response (Δ*I*_SC_ without *Ani9* 8.59 ± 1.75 *μ*A/cm²; with *Ani9* 6.14 ± 1.87 *μ*A/cm^2^; *P *=* *0.367, *n* = 5) nor the effect of CFTR_inh_172 (Δ*I*_SC_ without *Ani9* ‐5.58 ± 1.59 *μ*A/cm²; with *Ani9* ‐4.49 ± 1.54 *μ*A/cm^2^; *P *=* *0.636, *n* = 5) (Fig. 6D). The UTP‐induced *I*_SC_ transient in these experiments was reduced by *Ani9* from 9.0 ± 1.95 *μ*A/cm^2^ without *Ani9* to 0.17 ± 0.24 *μ*A/cm^2^ with *Ani9* (*P *<* *0.01, *n* = 5) (Fig. 5D). These experiments show that *Ani9* is particularly useful for studies of ANO1 in airway epithelia, as it compromises neither CFTR activity nor intracellular Ca^2+^ signaling.

## Discussion

**Figure 7:**
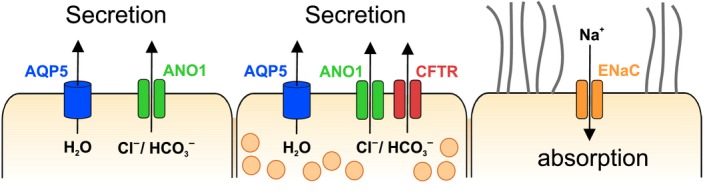
Schematic summary of the distribution of ion channels in rat tracheal epithelial cells. Our immunohistochemical results show that apical anion secretion and cation absorption do not occur in the same cells. Anions, water and mucins (symbolized by secretory vesicles) are secreted to the luminal ASL by secretory goblet cells *(center)*. Ciliated cells *(right)* promote mucociliary clearance using motile cilia, and also mediate Na^+^ absorption through ENaC proteins. A third cell type, probably representing club cells *(left)*, mediates Ca^2+^‐dependent Cl^−^ secretion through ANO1 channels.

The combination of immunohistochemical and electrophysiological methods in this study made it possible to postulate that ANO1 channels provide a Ca^2+^‐regulated Cl^−^ secretion pathway located in nonciliated tracheal epithelial cells. This pathway depends on intracellular Ca^2+^ signals elicited by secretagogues like the P2Y_2_ agonist UTP. The secretory response mediated by ANO1 channels is fast and transient, because the supply of releasable Ca^2+^ is limited. The high agonist concentrations used here (100 *μ*mol/L UTP, continuous application) exhausts intracellular Ca^2+^ stores within ~1 min and terminates UTP responses accordingly. Previous studies have shown that weaker or pulsatile P2Y_2_ stimulation by apical nucleotides may cause persistent Ca^2+^ oscillations both in secretory and in ciliated cells of the conducting airways. The result is an increased Cl^−^ secretion followed by improved hydration of the ASL and an increase in beat frequency in the ciliated cells (Evans and Sanderson 1999; Tarran et al. 2002; Warren et al. 2010). Together, these processes accelerate the mucociliary clearance at the airway surfaces (Mall 2008) and, hence, the removal of pathogens and other detrimental material from the lung. Our data show that, in rat, CFTR, ANO1, and AQP5 are expressed exclusively in nonciliated cells (Fig. 7).

CFTR and ANO1 may provide important control functions over the secretory activity in these cells, mediated through the cAMP‐ and the Ca^2+^‐ mediated intracellular signaling pathways. In addition to other Cl^−^ cannels, which support basal secretory activity, CFTR (Collawn and Matalon 2014) and ANO1 (Caputo et al. 2008; Lin et al. 2015) constitute pathways for cAMP‐ and Ca^2+^‐dependent regulation of secretion in nonciliated cells. Both channels can be activated by the system for autocrine and paracrine stimulation that uses the nucleotides ATP and adenosine as apical signals to accelerate mucociliary clearance. The nucleotides are released into the ASL (Okada et al. 2006; Button et al. 2013) and promote secretion of Cl^−^, water and mucins through P2Y_2_ receptors and, after metabolism of ATP to adenosine, through A_2b_ receptors (Lazarowski et al. 2004; Tarran et al. 2005). The nucleotide‐mediated signaling pathway also acts on ENaC which, in rat tracheal epithelium, appear to be exclusively expressed in ciliated cells. ENaC mediates Na^+^ absorption from the ASL and provides the electrical driving force for paracellular Cl^−^ flux from the ASL to the serosal fluid. The activity of ENaC is rate‐limiting for passive Cl^−^ and water absorption in tracheal epithelium and, therefore, a major determinant of ASL homeostasis (Matsui et al. 2000; Mall 2008). The purinergic system inhibits ENaC activity, thereby reducing the absorption of Na^+^, Cl^−^ and water and, hence, the depletion of the ASL layer (Mall et al. 2000; Kunzelmann et al. 2005). Thus, both nonciliated and ciliated cells in rat tracheal epithelium are critical components for ASL homeostasis and appear to regulate the properties of the thin liquid film that covers the tracheal surface antagonistically.

The presence of two different types of Cl^−^ channels in the same nonciliated cell can be interpreted in two ways. CFTR and ANO1 may be considered additive systems, with one channel providing continuous maintenance of the ASL, the other supplying a means to transiently boost secretion when necessary. Another view is that ANO1 may represent a failsafe system for stress situations, in particular for viral or bacterial insult that may compromise CFTR regulation but, at the same time, boost ANO1 activity (Caci et al. 2015). In any case, the two separate Cl^−^ channels of the airway secretory cell safeguard the vital function of ASL formation. Interestingly, in the human airway epithelia, the two channel types reside in separate cell types, as CFTR expression was localized together with ENaC in ciliated cells, while ANO1 was found to be restricted to nonciliated cells (Kreda et al. 2005; Scudieri et al. 2012). Colocalization of CFTR and ENaC in ciliated cells is considered to be crucial for ASL homeostasis in the human lung, because the *cftr*‐gene product can reduce ENaC activity, thus shifting the balance from absorption to secretion in ciliated cells (Kunzelmann et al. 2001; Gentzsch et al. 2010). The loss of the *cftr* control over ENaC activity in human cystic‐fibrosis patients induces Na^+^ hyperabsorption and promotes depletion of the ASL, a phenomenon not observed in CFTR^−/−^ mouse models (Grubb and Boucher 1999). Our finding that CFTR and ENaC are expressed in different cells types of the rat tracheal epithelium is in accordance with this observation. The separate expression of CFTR and ENaC makes a direct interaction and regulatory relationship between these proteins unlikely in rat lower airways. However, separation of CFTR and ENaC does not necessarily impede a coordinated regulation of the two pathways. Purinergic regulation may activate CFTR and ANO1 in nonciliated cells and, at the same time, inhibit ENaC in ciliated cells. Moreover, as reported earlier, Ca^2+^‐dependent secretion plays a more prominent role in mouse airway epithelia than in mouse intestinal epithelia (Clarke et al. 1994). The colocalization of ANO1 with CFTR in nonciliated cells is consistent with a maintenance of ANO1‐mediated Cl^−^ secretion in CFTR^−/−^ animals. In a similar way, rat tracheal epithelium appears to rely on ANO1, more so than the human airways.

The use of RTEC cultures for our Ussing‐chamber experiments was based on previous characterizations of primary rat tracheal epithelial cultures (Hwang et al. 1996). RTEC cultures were shown to generate ENaC‐mediated and CFTR‐mediated transepithelial currents, and their response to apically applied UTP and ATP involved P2Y_2_ receptors and Ca^2+^‐dependent Cl^−^ channels. RTEC cultures form polarized cell monolayers that consist of ciliated and nonciliated cells, joint by tight junctions. Although not identical to native tracheal epithelium, this preparation is suitable to examine components of the transepithelial current. In this study, we found pharmacological evidence that the Ca^2+^‐dependent Cl^−^ channels that mediate UTP‐stimulated Cl^−^ secretion in RTEC cultures are ANO1 proteins. This result is consistent with earlier studies from new‐born ANO1‐knockout mice where the UTP response of native tracheal tissue was strongly reduced, but not completely suppressed (Ousingsawat et al. 2009; Rock et al. 2009). It is possible that the native UTP response contains a second component, still present in the newborn ANO1‐knockout mouse, which is not present in our recordings from RTEC cultures. The almost complete suppression of the UTP response by the ANO1‐blocker *Ani9* in our experiments suggests that only ANO1 channels open in RTEC cultures when the intracellular Ca^2+^ concentration increases.

In our histological studies, we faced limitations mainly with regard to the classification of epithelial cells. While ciliated cells could be identified with some certainty, the characterization of nonciliated cells relied on Muc5b expression. In human and mouse tracheal epithelia, the gel‐forming mucin MUC5B/Muc5b is expressed in goblet cells (Groneberg et al. 2002) and we, therefore, used Muc5b as a *bona fide* marker for goblet cells. However, a subset of nonciliated cells was Muc5b‐negative, but expressed ANO1. These cells may represent club cells (Tokita et al. 2014), but they still have to be properly characterized. An unambiguous mapping of CFTR‐ and ANO1‐ expression to the various sub‐populations of nonciliated cells in the rat airways awaits further histological examination. Our study focused on the ion channels depicted in Fig. 7, and did not address contributions of other transport proteins to Cl^−^ secretion in the trachea. However, various other Cl^−^ channels and transporters may be involved in the secretory process and may play crucial roles in the regulation of Cl^−^ transport in the rat tracheal epithelium (Sala‐Rabanal et al. 2015a). Among the most interesting candidates are the SLC26A9 protein as a constitutively active Cl^−^ channel in airway epithelia (Bertrand et al. 2009; Anagnostopoulou et al. 2012; Salomon et al. 2016), the CLCA1 protein as a modifier of ANO1 activity (Sala‐Rabanal et al. 2015b), and SLC26A4/pendrin as an epithelial Cl^−^/HCO_3_^−^ exchanger (Lee et al. 2015). The individual expression patterns of these proteins in ciliated and nonciliated cells, as well as their contributions to basal and nucleotide‐stimulated Cl^−^ transport, will be an important topic of future studies of the rat airway epithelia.

Taken together, our results demonstrate that the Cl^−^ channels CFTR and ANO1 are largely, but not always, coexpressed with AQP5 and Muc5b in nonciliated cells of the rat tracheal epithelium. These cells thus provide a cellular pathway for Cl^−^, water and mucin secretion. In contrast, ciliated cells express ENaC, but neither AQP5 nor CFTR or ANO1, and are equipped for Na^+^ absorption which can drive paracellular Cl^−^ and water uptake from the luminal fluid. The functional characterization of ANO1 was achieved using the blocker *Ani9* that displays selectivity for this Ca^2+^‐activated Cl^−^ channel without interfering with CFTR or cellular Ca^2+^ signaling. The almost total block of the UTP‐induced Δ*I*_SC_ signal suggests that ANO1 channels mediate this secretory response.

## Conflict of Interest

The authors disclose no conflict of interest.

## Data Accessibility

## Supporting information




**Fig S1.** Representative control experiments for ANO1 immunohistochemistry.
