# Supplementary material for: Cellular distribution and function of ion channels involved in transport processes in rat tracheal epithelium
Source: Physiol Rep. 2017 Jun 22;5(12):e13290. doi: 10.14814/phy2.13290 (PMC5492199; doi:10.14814/phy2.13290)
Supplement: Supplementary file 1 — Fig S1. Representative control experiments for ANO1 immunohistochemistry. [file PHY2-5-e13290-s001.docx]

**Supplementary Figure S1** Representative control experiments for ANO1 immunohistochemistry. (A, B) Non-transfected HEK293 cells are not stained after overnight incubation with rabbit α-ANO1 and goat α-ANO1 primary antisera with donkey α-rabbit 568 and donkey α-goat 568 secondary antibodies. The blue signal is DAPI nuclear stain. (C, D) HEK293 cells transfected with an ANO1-YFP fusion protein display YFP fluorescence. In the absence of a primary antiserum they do not display signals of the secondary antisera donkey α-rabbit 568 and donkey α-goat 568. (E, F) In the presence of the primary antisera rabbit α-ANO1 or goat α-ANO1, the fluorescence of secondary antisera donkey α-rabbit 568 and donkey α-goat 568 merges with the ANO1-YFP signal. (G) Control of secondary antisera donkey α-rabbit 488 and goat α-mouse 568 on a section of rat tracheal epithelium without primary α-ANO1 antiserum. The blue signal is DAPI nuclear stain. Calibration bars are 5 µm (A-F) and 20 µm (G).
